# Noodles Made from High Amylose Wheat Flour Attenuate Postprandial Glycaemia in Healthy Adults

**DOI:** 10.3390/nu12082171

**Published:** 2020-07-22

**Authors:** Kim Ang, Carla Bourgy, Haelee Fenton, Ahmed Regina, Marcus Newberry, Dean Diepeveen, Domenico Lafiandra, Sara Grafenauer, Wendy Hunt, Vicky Solah

**Affiliations:** 1 School Public Health, Curtin University, Perth 6845, Western Australia, Australia; kimang03@yahoo.com.au (K.A.); carla.bourgy@blackmores.com.au (C.B.); h.fenton@curtin.edu.au (H.F.); wendy.hunt@aegic.org.au (W.H.); 2 School of Molecular and Life Sciences, Curtin University, Perth 6845, Western Australia, Australia; 3 Commonwealth Scientific and Industrial Research Organisation, Agriculture and Food, Canberra 2601, ACT, Australia; a.regina@irri.org (A.R.); marcus.newberry@csiro.au (M.N.); 4 Department of Primary Industries and Regional Development, South Perth 6151, Western Australia; Australia; dean.diepeveen@dpird.wa.gov.au; 5 College of Science, Health, Engineering and Education, Murdoch University, Murdoch 6150, Western Australia, Australia; 6 Department Agricultural and Forestry Sciences, University of Tuscia, 01100 Viterbo, Italy; lafiandr@unitus.it; 7 Grains and Legumes Nutrition Council, North Ryde 2113, Australia; sarag@glnc.org.au; 8 Australian Export Grains Innovation Centre, South Perth 6151, Western Australia, Australia

**Keywords:** blood glucose, glycaemic response, resistant starch

## Abstract

Previous research has not considered the effect of high amylose wheat noodles on postprandial glycaemia. The aim of the study is to investigate the effect of consumption of high amylose noodles on postprandial glycaemia over 2-h periods by monitoring changes in blood glucose concentration and calculating the total area under the blood glucose concentration curve. Twelve healthy young adults were recruited to a repeated measure randomised, single-blinded crossover trial to compare the effect of consuming noodles (180 g) containing 15%, 20% and 45% amylose on postprandial glycaemia. Fasting blood glucose concentrations were taken via finger-prick blood samples. Postprandial blood glucose concentrations were taken at 15, 30, 45, 60, 90 and 120 min. Subjects consuming high amylose noodles made with flour containing 45% amylose had significantly lower blood glucose concentration at 15, 30 and 45 min (5.5 ± 0.11, 6.1 ± 0.11 and 5.6 ± 0.11 mmol/L; *p* = 0.01) compared to subjects consuming low amylose noodles with 15% amylose (5.8 ± 0.12, 6.6 ± 0.12 and 5.9 ± 0.12 mmol/L). The total area under the blood glucose concentration curve after consumption of high amylose noodles with 45% amylose was 640.4 ± 9.49 mmol/L/min, 3.4% lower than consumption of low amylose noodles with 15% amylose (662.9 ± 9.49 mmol/L/min), *p* = 0.021. Noodles made from high amylose wheat flour attenuate postprandial glycaemia in healthy young adults, as characterised by the significantly lower blood glucose concentration and a 3.4% reduction in glycaemic response.

## 1. Introduction

Noodles, originating in China, are traditionally a staple food in the Asian population [1] but have become a popular food product worldwide [2]. There is a vast range of wheat-based Asian noodles that are commonly made from refined wheat flour but vary with respect to formulation, size and shape, and processing method [3]. Consumption of some types of noodles, such as instant noodles, may adversely affect health. A study by Huh, Kim and Jo et al. [4] suggested that high and frequent consumption of instant noodles is associated with the increased risk of diet-related diseases such type-2 diabetes mellitus (T2DM).

The worldwide prevalence of diabetes is projected to rise rapidly from 382 million people in 2013 to an estimated 592 million by 2035 [5]. Insulin resistance [6] and oxidative stress [7] caused by regular episodes of pronounced fluctuations in postprandial glycaemia (PPG) have been identified as important factors contributing to the onset of T2DM [8,9,10,11,12]. Postprandial glycaemia may be influenced by the type and amount of carbohydrates consumed [12,13,14,15,16], starch interaction with other components in the meal [17,18], physical form of the food [6,19], food particle size [12,20], amylose to amylopectin ratio [18,21,22] and the capacity of individuals to uptake glucose via insulin-induced glucose utilisation [6]. Dietary modification is a known strategy to assist in modulating PPG in the management and mitigation of risk factors associated with T2DM [7,23].

Starch is a glucose polymer comprising of amylose—primarily a linear chain linked together by α-1, 4-glycosidic bonds—and amylopectin—a highly branched chain with α-1, 6-glycosidic bonds [18]. In wheat, starch constitutes 65–75% of the dry grain weight, with amylose and amylopectin content in the starch ranging between 20–30% and 70–80% respectively [18]. Wheat containing more than 28% amylose has been previously categorised as high amylose [3] but Hallstrom et al. [18] reported on research using wheat with 38% amylose. In recent years, researchers have developed a wheat variety with a starch amylose content of 85% [22].

Evidence from previous studies examining the consumption of high amylose (HA) food products such as smoothies, snack bars, cookies [24,25], muffins [26], breakfast cereal [27], bread [18,28], pasta [19], and rice [29,30] have found attenuation in PPG, where a lower blood glucose level and reduction in glycaemic response (GR) were observed. However, the degree of attenuation in PPG varied between studies. Studies have also confirmed that the type and form of food containing the amylose [19,31], the source and cultivar type [30,32], and how the food is prepared [33] are other factors responsible for the reported differences in the attenuation of PPG between foods.

Therefore, this study (P1) used HA noodles as an alternative food form to investigate the effect of HA on PPG. Moreover, noodles made from HA wheat flour had not been previously considered. The study aims to examine the effect of consuming HA noodles on PPG by firstly monitoring changes in the blood glucose concentration postprandially at different time points over 2-h periods. Secondly, to compare the glycaemic response (GR) by calculating the total area under the blood glucose concentration curve (AUC). It was hypothesised that consumption of HA noodles would attenuate PPG by reducing blood glucose concentration and moderate GR.

## 2. Materials and Methods

**Figure 1:**
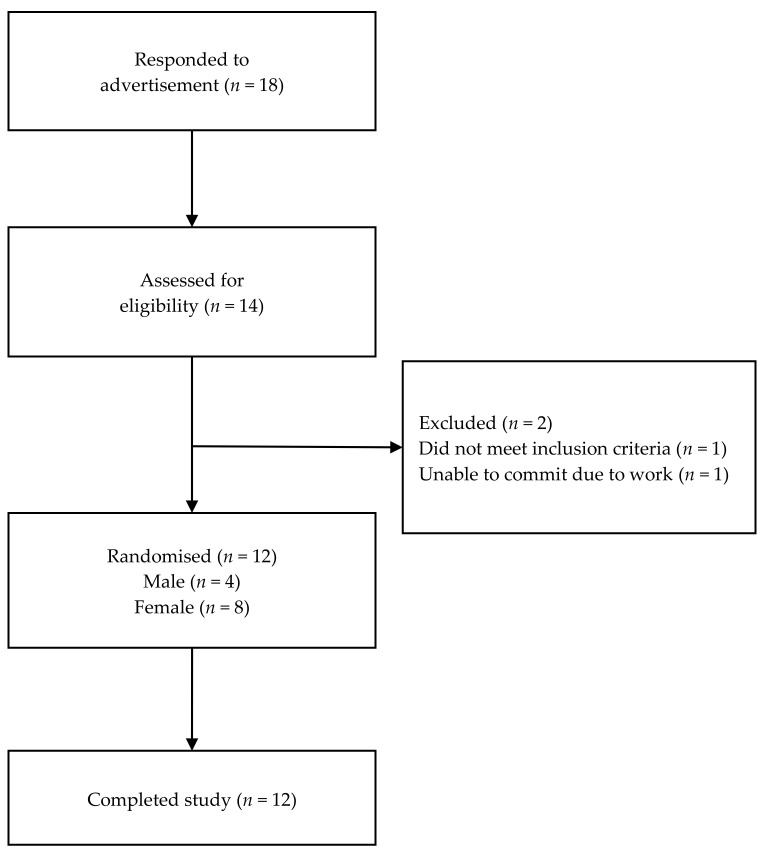
Flow diagram describing the progress of the subjects through study (P1).

Young, healthy male and female adults with body mass index between 18.3 to 23.5 kg/m^2^, aged 20 to 26 years, were recruited via social media posts and flyers placed around the campus of Curtin University, Perth, Western Australia. Subjects were excluded if they had diabetes, cardiovascular disease or metabolic disorders, were a smoker, pregnant or lactating, allergic to the test meals, on weight loss programs, taking appetite suppressants or medication known to affect blood glucose, and/or consumed more than three standard drinks of alcohol a day. A screening questionnaire (Appendix A) was used to assess eligible subjects against these criteria. Subjects’ eating habits were assessed using three-factor eating questionnaires [34]. The Human Research Ethics Committee of Curtin University approved the current study (HRE2017-0467). Informed written consent was obtained from all subjects prior to commencement of the study. Subjects were given gift vouchers as tokens of appreciation for their participation. The progress of the subjects through the study is presented in Figure 1. 

A repeated measure randomised, single-blinded crossover trial was used to compare the effect of PPG following the consumption of three types of noodle test meals: low amylose noodles containing 15% (LAN15) and 20% (LAN20) amylose, and high amylose noodles containing 45% (HAN45) amylose, as shown in Table 1.

Subjects were served freshly prepared 180 g noodle meals at weekly intervals, with blood glucose measurements taken over 2-h periods following each meal. The day before each testing day, subjects were asked to refrain from drinking alcohol and instructed to eat a standardised dinner selected from a choice of three (On The Menu: Roast Beef, Roast Lamb or Roast Chicken, Vesco Foods Pty. Ltd., Osborne Park, Australia), accompanied by a nut bar (Nice & Natural Chocolate Nut Bar, Nice & Natural, Auckland, New Zealand), crispbread (Premium Wheat Bran Crispbread, Mondelez Australia Biscuits, Victoria, Australia), juice (Just Juice Tropical Punch, The Daily Drink Co., Victoria, Australia) and/or cheese (Cracker Barrel Extra Sharp, The Warrnambool Cheese & Butter Factory Company Ltd., Victoria, Australia) to meet their individual energy (kJ) needs, which were controlled based on their usual energy intake.

On the first visit, subjects were randomly assigned a three-digit number using an online random number generator (www.randomization.com), with each number corresponding to a unique test meal sequence. On test days, subjects arrived at 08:00 a.m. to Building 400, Curtin University, having fasted for 10 h overnight and consumed only water ad libitum. In the clinic room, instructions on the testing protocol were given. Subjects’ weight and height were measured in light clothing without shoes. Baseline finger-prick blood samples were taken before test meals to measure fasting blood glucose.

Subjects were then seated in the sensory booth where the test meals were served. Subjects consumed each test meal twice: two each of LAN15, LAN20 and HAN45 in random order over a 6-week period. Subjects were instructed to consume the 180 g test meals within 12 min. After consuming the test meals, subjects did not eat or drink until the last finger prick was taken at 120 min post-meal. They were moved to an adjacent room where they could read or work on a computer while waiting for the appointed time for blood glucose measurement. Postprandial blood glucose concentrations were measured at 15, 30, 45, 60, 90 and 120 min.

This study (P1) followed a preliminary study involving a panel of 11 subjects (S1). In the S1 preliminary study, the difficulty in processing HAN60 flour to noodles often resulted in an inconsistent product that needed to be reprocessed. As a result, the HAN45 was selected as the high amylose flour to allow a standardised process. LAN15 and LAN 20 were milled by the Australian Export Grain Innovation Centre, Perth, Australia, from wheat grown in Shackleton, Western Australia, for the study (P1). The high amylose flour (71% amylose) and low amylose flour (LAN27) (S1) were milled from wheat grown by Australian Grain Technologies (AGT) in Wagga Wagga, Australia, and provided by the Commonwealth Scientific and Industrial Research Organisation for the study. HAN45 and HAN60 (S1) were a blend of the high amylose flour (71% amylose) and the LAN27 flour. LAN15, LAN20 and LAN27 (S1) flours were used “as is”, i.e., not blended before being made into noodles.

Moisture was determined using AOAC (Association of Official Agricultural Chemists)International Method 925.098 (105 °C for 24 h). Total ash and protein (*N* × 5.7) were determined using AACCI Methods 08-01.01 and 46-12.01. The total starch content was determined using a Megazyme total starch assay kit (without DMSO) using AOAC International Method 996.11. Resistant starch was determined using the Megazyme AOAC International Method 2002.02. The apparent amylose content of starch in all flours used to make noodles, including the two blended flours, was determined using a Megazyme amylose/amylopectin kit (K-AMYL) that employs concanavalin A (Megazyme International, Ltd., Bray Co., Wicklow, Ireland). Pasting properties of starch were determined using a Rapid Visco-Analyser employing AACCI Method 76-21.02.

The noodles were made using 500 g of the wheat flour consisting of 15%, 20%, 45% amylose with 160 mL, 170 mL and 180 mL of water added to the respective batches of flour. The additional water needed to produce a consistent crumb was consistent with the higher Farinograph water absorption observed in the 20% and 45% amylose wheat flour (Table 2). The flour and water were combined and mixed into a consistent crumb using a Philips Pasta and Noodle Maker pin mixer (Philips Pasta and Noodle Maker HR235706, China). After 3 min of mixing, the dough was extruded using the Philips noodle mould (Philips Pasta and Noodle Maker HR235706, China). The raw noodles were cooked for 5 min and left to rest. The higher water absorption with increasing amylose found on mixing was not found during boiling as the weight increase (weight increase (g)/weight of raw noodles (g) × 100) from fresh to cooked for LAN15 was 50% whereas LAN20 was 44% and HAN45 was 34%. Prior to consumption, the noodles were reheated in boiling water for 2 min and served warm with 20 mL of soy sauce and a 250 mL glass of water.

Seven finger-prick capillary blood samples were collected from each subject using a HemoCue Glucose RT microcuvette (HemoCue Australia Pty Ltd., Wamberal, Australia). Capillary blood glucose was measured using a HemoCue Glucose 201 RT analyser (HemoCue Australia Pty Ltd., Wamberal, Australia).

Data were gathered from duplicate testing of LAN15, LAN20, HAN45 (P1) meals. All statistical analyses were performed using Genstat for Windows, release 18.1 (VSN International Ltd., Hemel Hempstead, UK). Data were assessed for normality and expressed as means ± standard error of the mean (SEM). Restricted maximum likelihood (REML) variance components analysis was used to evaluate the interactions between test meal, time and subjects on blood glucose. Differences in testing days were adjusted for in the analysis. Means of blood glucose concentrations at each time point were generated to calculate the area under the curve (AUC). The blood glucose AUC was calculated using the trapezoidal rule [35] to quantify the glycaemic response (GR) to the test meals. Analysis of variance (ANOVA) was used to identify significant differences between blood glucose AUC at different time periods. The sample size was set at 12 for 80% power at a level of *p* < 0.05, based on the recommendation by Brouns et al. [36] for statistical significance.

## 3. Results

In the study (P1), 18 subjects responded to the advertisement, with 12 subjects matching the selection criteria. All 12 subjects completed the study. Subjects’ baseline characteristics are shown in Table 3.

**Figure 2:**
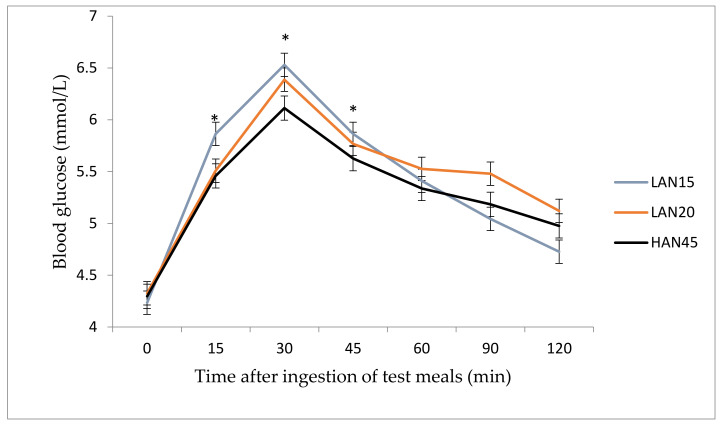
P1 study: The effects of consuming noodles containing 15%, 20% and 45% amylose on postprandial glycaemia at different time periods over 120 min. High amylose noodles (45%) induced significantly lower blood glucose at 15, 30 and 45 min (*p* = 0.027). Values presented as mean ± SEM. * indicates statistical significance (*p <* 0.05).

Postprandial glycaemia over 120 min was affected by time (*p* < 0.001), test meal (*p* = 0.027) and subjects (*p* = 0.010). The postprandial blood glucose concentrations for each of the three test meals are depicted in Figure 2. Following the consumption of LAN15, LAN20 and HAN45 test meals, blood glucose concentrations peaked concurrently at 30 min, with a significantly lower peak observed for HAN45 (6.1 ± 0.11 mmol/L, *p* = 0.027) compared to LAN15 (6.5 ± 0.12 mmol/L) and LAN20 (6.3 ± 0.12 mmol/L). Statistically significant lower blood glucose concentrations were also observed for HAN45 at 15, 30, and 45 min (*p* = 0.027). At 120 min, a sharp decline in PPG was observed for LAN15 (4.7 ± 0.12 mmol/L, *p* = 0.027) compared to LAN20 (5.1 ± 0.11 mmol/L) and HAN45 (5.0 ± 0.11 mmol/L). Continued steady declines were observed for LAN20 and HAN45.

**Figure 3:**
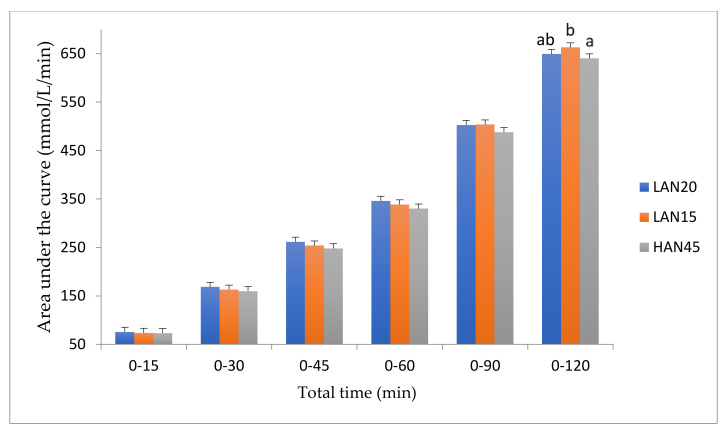
P1 study: Area under the blood glucose curve over 120 min following the ingestion of test noodles containing 15%, 20% and 45% amylose. Blood glucose total area under the curve for high amylose noodles (45%) was significantly lower compared to low amylose noodles (15%) (*p* = 0.021). Data presented as mean ± SEM.

The cumulative blood glucose AUC was the lowest for HAN45 over all time periods (Figure 3). The total blood glucose AUC for HAN45 over 120 min was significantly lower (640.4 ± 9.49 mmol/L/min, *p* = 0.021) compared to LAN15 (662.4 ± 9.49 mmol/L/min), resulting in a statistically significant reduction of 3.4% in GR following the consumption of HAN45 (*p* = 0.021). High amylose noodles with 45% amylose produced the least amount of fluctuation in PPG, as compared to LAN15 and LAN20, due to the smaller peak at 30 min and steady moderated decline in blood glucose concentration thereafter.

## 4. Discussion

This research (P1) revealed a lower peak and moderated subsequent decline in blood glucose level following the consumption of HAN45 noodles in healthy volunteers. This phenomenon is very likely associated with the location and distribution of amylose in relation to amylopectin in starch granules. Although presently not well understood, it is believed that the way these components are organised gives rise to considerable variability in the properties of starch granules between and within species [22,37]. It has been hypothesised that the bulk of large, long and compact amylose chains are concentrated within the granule core, with smaller quantities of short amylose chains interspersed with amylopectin clusters located towards the periphery [21,22,37].

The large, long compact structure of amylose that is abundant in the core is slow to gelatinise on cooking and quick to retrograde on cooling [18,37]. Amylose has extensive hydrogen bonds, which require more energy to break [38]. Therefore, the gelatinisation temperature increases with higher amylose content [39]. This contributes to reduced gelatinisation [39], impeding enzyme accessibility [18], which then reduces the rate of digestion, resulting in lowered peak PPG at 30 min followed by a gradual and moderated decline in blood glucose concentrations thereafter.

Furthermore, a high proportion of short amylose chains are interspersed with amylopectin clusters at the periphery of the starch granule in high amylose starch. This will render a hard shell on the surface of the starch granule [22,37], which increases the resistance of starch to digestion. As enzymatic hydrolysis is hindered [37], the reduced rate of digestion of high amylose noodles led to the statistically significant lowered postprandial blood glucose concentrations at 15, 30 and 45 min and a 3.4% reduction in GR when compared with low amylose noodles, which is in line with the findings of previous studies. However, the degree of reduction is small compared to previous studies on bread that reported at least 17% reduction in GR [18]. There is a large body of knowledge [37,40] showing that HA content is not the only factor influencing the attenuation of PPG. The source or cultivar of amylose [26,32], the amount of resistant starch [18,28], type of food, amount and composition of food [11,41], as well as processing method [42,43] were considered to be equally important when assessing its impact on PPG.

Keogh et al. [26] and King et al. [27] found that HA barley cultivar reduced GR by 22% and 35% following the consumption of HA barley cereal compared to low amylose wheat cereal and barley cereal, respectively. O’Connor et al. [24] found 34% and 37% reductions in GR after consuming a beverage and a snack bar containing HA maize co-processed with guar gum. In addition to being high in amylose, these HA food products contained either high beta-glucan or guar gum. Guar gum and beta-glucan form soluble viscous fibres that limit the interaction of digesta with digestive enzymes, thus reducing the rate of gastric emptying and the rate of digestion, thereby increasing the reduction of GR [15,44]. Soluble viscous fibres also restrict starch gelatinisation by limiting the availability of water for starch hydration [15,44]. The combination of HA and soluble viscous fibre further limits starch gelatinisation [45], which is in line with the claim that the degree of starch gelatinisation is inversely related to the reduction in GR [46].

Cooking and different processing methods influence the physical properties and chemical structure of foods [42,47]. The effects of cooking [18,33] and low storage temperature [40] have been reported to increase the formation of retrograded amylose, a resistant starch type 3 (RS3) that forms during the cooling of gelatinised starch [38]. Higher yields in RS3 are concomitant with higher amylose content [18,33]. Hasjim [28] and Hallstrom [18] found 55% and 28% reductions in GR, respectively, following the consumption of HA bread. The former containing 37.4 g of RS3 compared to 6.5 g for control [28], and the latter containing 19% of RS3 compared to 8.7% for control [18]. This corresponds with a study by Lin [48], where an increased reduction in GR was seen when RS3 was used to substitute 10%, 30% and 60% of white wheat flour in bread. The high proportion of RS3 increased the degree of enzyme resistance, which reduced the rate of digestion [40,49], leading to greatly increased GR attenuation. However, not all studies [29,33] have shown a reduction in GR after RS consumption [50]. The inconsistency in the findings may be attributed to the differences in food type [51], as well as differences in the sources and the amounts of RS [52].

In a study by Banchathanakij et al. [22], the consumption of HA bread made with flour containing 74% amylose, water, oil, sugar, salt, yeast, and bread improver resulted in a 39% lower glycaemic AUC response compared to the consumption of low amylose wheat bread. The reduction in GR to the HA bread was most pronounced at 30 min in this study. Participants consumed 121 g servings of bread containing 50 g carbohydrate (sugar plus starch) in the low amylose bread, whereas 30 g of carbohydrate (sugar plus starch) was consumed in the HA bread.

Unlike previous studies, the noodles in the present study (P1) were made from wheat flour with only water added. The noodles were made and eaten fresh hence limiting the potential formation of RS3, described in a study by Chiu and Stewart [33], where less RS3 content was found in fresh rice compared to refrigerated rice. The elimination of factors such as the difference in food composition, the high proportion of RS3 and low storage temperature add to the uniqueness of this study as it considers the effect of differences in amylose in isolation. This would justify the small 3.4% reduction (*p* = 0.021) in GR in the present study as compared to at least 17% reduction [18] in previous studies.

Studies [18,27,30] considering HA food products have mostly found evidence of attenuation in PPG, which supports the findings of the present (P1) study. Noodles are traditionally made from refined wheat flour containing approximately 17–20% amylose [3]. Food products made from refined wheat flour with low amylose content are associated with a faster rate of digestion [14] and elevated peaks in PPG [9]. Consumption of wheat-based food, including noodles, in the Hong Kong and Singaporean Chinese populations have been associated with significantly higher risks of T2DM [1,53], whereas a similar study of a Japanese cohort was not associated with the risk of T2DM [54]. The difference may be due to the type of noodles consumed [54] and the increased consumption of dumplings, bread, cakes, pancakes, and Rou Jia Mo (Chinese hamburger bread) [55], whereas whole-grain buckwheat noodles are commonly consumed in Japan [54]. Therefore, it is beneficial to use HA wheat flour in commonly eaten foods, such as noodles, to reduce fluctuations in PPG, which can mitigate the risk of developing T2DM.

After accounting for the compounding effects of differences in food composition, such as fibre and RS in HA food products that could potentially contribute to the attenuation in PPG [18,44], the positive effects of HA noodles on PPG remains promising. However, the ability of this study to unequivocally conclude the direct effect of amylose on PPG was complicated by the variations in the protein content of the wheat flour samples (10.1% to 15%) used. Protein can be a factor that affects starch digestibility and, consequently, PPG [56]. Wheat proteins tend to form resilient gluten networks that entrap starch granules, hence reducing the accessibility of digestive enzymes to the starch granules [56].

This study (P1) was part of a larger study involving a previously unpublished preliminary study on the direct effect of HA on PPG (S1). In a preliminary meal-test study (S1), eleven healthy young adults were recruited to a repeated measure randomised, single-blinded crossover trial to compare the effect of consuming noodles containing 27% (LAN27) and 60% amylose (HAN60) on postprandial glycaemia (Table S1). Subjects in the S1 study (Table S2) consumed 180 g noodles after a 10-h fast although the evening meal was not controlled as in the P1 study. Six subjects were common to both studies, with the remaining five demonstrating similar characteristics to the subjects who participated in the current study (P1; Table 3). Test noodle meals in the S1 study had a constant protein content of 16.2% in low amylose noodles with 27% amylose (LAN27) and high amylose noodles with 60% amylose (HAN60). Analysis of the collected data showed that HAN60 produced significantly lower postprandial blood glucose concentration at 45, 60 and 90 min compared to LAN27 (Figure S1). The statistically significant difference *(p <* 0.001) in the blood glucose concentrations in the S1 study suggested that despite the compounding effects of other components found in HA food products, a positive effect of HA food products on PPG can be concluded. A secondary analysis was also carried out, combining results from P1 and S1 in order to standardise for amylose and solely examine the effect of protein. The results indicated that a protein content difference of 4.9% (HAN45–LAN15) had no effect on postprandial glycaemia (*p* > 0.05) for the study (P1), but more research is needed as higher protein plus higher amylose may have a synergistic effect on postprandial glycaemia. A review by Paterson et al. [57] supports the limited effect of small increases in protein in carbohydrate-containing meals and reports an increase of ≥ 12.5 g of protein when consumed with 30 g of carbohydrate is needed for an impact on postprandial glycaemia. Even though the content of amylose increased with protein content in the current study, it can be deduced that the impact on PPG is directly linked to HA content, as supported by secondary analysis.

The blood glucose AUC in the S1 study was lowest for HAN60 over all time periods, similar to HAN45 in the present (P1) study. Total blood glucose AUC for HAN60 was significantly lower (*p* = 0.009) than LAN27 in the study (Figure S2). Likewise, for the present study (P1), total blood glucose AUC for HAN45 was also significantly lower (*p* = 0.021) than LAN15. High amylose noodles with 60% amylose induced a 6.5% reduction in GR, while HAN45 reduced GR by 3.4%. The difference in the degree of reduction can be attributed to increased amylose content contributing to a reduction of available carbohydrate. Given the consistency in the results, it can be concluded that attenuations in PPG were largely due to HA content.

The strengths of this study include the collection of data from duplicate testing, hence increasing the precision of data collected, and the 6-day washout period that eliminated the risk of interference from previous treatments. However, there are a number of limitations that require acknowledgement. The protein content of the wheat flour samples used was not controlled for in the current study because suitable samples were not available at the time of testing. Protein is believed to moderate starch digestion, hence influencing PPG [56]. However, based on the S1 study, where protein was controlled for and produced analogous results, it can be deduced that HA in the wheat noodles used in this study was a significant factor contributing to the attenuated PPG. Future studies using controlled wheat flour samples with variations in amylose content and the same protein content would further strengthen the validation of the effect of HA wheat noodles on PPG.

Flour blending is a common method used to obtain the desired amount of amylose content in flour samples. Whilst it changes the amylose composition of the flour sample to suit the purpose of the research, the blending process has the potential to introduce variations in other components such as protein, fat and fibre. Future studies could use wheat flour samples that are naturally grown with different percentages in amylose content. Obviating the need for blending can potentially reduce the likelihood of introducing uncontrolled variations of other components that may be found in the blended flour. Therefore, the direct effect of amylose on PPG can be validated with greater accuracy.

In the present study (P1), equal serve sizes of noodles by weight (180 g) were given to subjects for all test meals. Consequently, the intrinsic amount of available carbohydrate is thought to vary depending on the percentage of amylose content. The differences in the amount of available carbohydrate could have influenced PPG [14]. The attenuation of postprandial glycemia in the present study is likely due to the reduced amount and availability of carbohydrate. Future research on noodles with equal amounts of available carbohydrate in test meals should help confirm that attenuation of PPG is possible by replacing low-amylose noodles with higher-amylose noodles. In addition, the amylose and amylopectin chain length impact on the swelling ability of starch and the subsequent effect on PPG, described by Bhattarai et al. [58], is an important area of future research.

Even though statistical significance was observed in the reduction of GR, the small quantum of the reduction (3.4%) could be due to the small sample (*n* = 12) size. As a consequence, this result may not be adequate to exhibit clinical significance. Future studies increase the sample size to achieve greater precision and confidence in the result for clinical purposes.

Taking into consideration the preliminary study (S1) and previous studies, this study validates the plausibility of using HA noodles to assist glycaemic control in young, healthy individuals. Notwithstanding, the findings of this study cannot be generalised to overweight, obese and impaired glucose tolerance individuals as these subjects are likely to exhibit different metabolic responses. Future studies could investigate the effect of HA noodles on such subjects.

## 5. Conclusions

In conclusion, the present study has demonstrated that HAN45 made from HA wheat significantly reduced blood glucose concentrations at different time points and total blood glucose AUC showed a statistically significant reduction of 3.4% in glycaemic response. Even though there were variations in the protein content, the results of this study are consistent and were supported by the S1 study and secondary analysis. There is a strong link between attenuation in PPG and the consumption of food products containing HA. Collectively, there is strong evidence to support the hypothesis that consumption of HA noodles attenuate PPG. This finding can assist in mitigating the risk of developing diet-related diseases such as T2DM through diet modification, with the introduction of HA noodles as part of a well-balanced nutritious diet.

## Figures and Tables

**Table 1:** Wheat flour composition (P1) study.

Noodle Type	Moisture %	Protein %	Ash %	Starch (RS Not Included) %	Resistant Starch % of Flour	Amylose % of Total Starch
LAN15	11.8 (0.2)	10.1 (0.21)	0.31 (0.01)	80.3 (0.7)	-	15.0 (0.8)
LAN20	12.6 (0.2)	13.2 (0.35)	0.36 (0.03)	74.0 (1.0)	-	19.6 (1.5)
HAN45	12.0 (0.3)	15.0 (0.12)	0.48 (0.02)	66.1 (0.5)	* 8.8	45.1 (1.5)

Values are dry weight basis and the mean of duplicates and values in brackets are SD. RS, resistant starch. * calculated from LAN27 and HAN60 results. LAN, low amylose noodles; HAN, high amylose noodles.

**Table 2:** Wheat flour quality characteristics.

Noodle Type	RVA peak Viscosity (cP)	RVA Breakdown (cP)	RVA Final Viscosity (cP)
LAN15	3435	1635	1352
LAN20	3409	1395	1474
HAN45	787	309	866

RVA, Rapid Visco-Analyser; cP, centipoise

**Table 3:** Baseline characteristics of the subjects in P1 study ^(a).^

Characteristic	Total (*n* = 12)	Women (*n* = 8)	Men (*n* = 4)
Age (years)	22.25 ± 1.86	22.25 ± 1.67	22.25 ± 2.50
Weight (kg)	59.04 ± 11.88	52.81 ± 5.57	71.50 ± 11.62
Height (m)	1.68 ± 12.91	1.61 ± 4.11	1.83 ± 11.69
Body mass index (kg/m^2^)	20.63 ± 1.64	20.32 ± 1.28	21.25 ± 2.29
Fasting blood glucose (mmol/L)	4.30 ± 0.49	4.20 ± 0.51	4.50 ± 0.39

^(a)^ Data are expressed as mean ± SEM.

## Supplementary Materials

The following are available online at https://www.mdpi.com/2072-6643/12/8/2171/s1, Figure S1: S1 study- The effects of consuming noodles containing 27.2% and 60% amylose on postprandial glycaemia at different time periods over 120 min. High amylose noodles 60% induced significantly lower blood glucose at 45, 60 and 90 min (*p* < 0.001). Values presented as mean ± SEM, Figure S2: S1 study- Area under the blood glucose curve over 120 min following the ingestion of test noodles containing 27.2% and 60% amylose. Blood glucose total area under the curve for high amylose noodles 60% was significantly lower at 90 and 120 min compared to low amylose noodles 27.2% (*p* = 0.021). Data presented as mean ± SEM. Difference letters indicate a statistically significant difference *p* < 0.05, Table S1: Wheat flour composition (S1), Table S2: Characteristics of the 11 subjects (women = 10, men = 1) in S1.

## Appendix A

Screening Questionnaire

Participant Code:

(1) Height: cm.

(2) Weight: kg.

(3) Do you smoke? (Please tick either one)

Yes; No.

(4) Will you be willing to avoid consuming alcohol the day before and on the day of testing?

Yes; No.

(5) How many days a week do you normally have breakfast? (Please tick in the respective box for your answer, e.g., if your answer is “Never” please tick in the box below “Never”)

**Frequency of breakfast consumption**	**Never**	**Less than once a week**	**1–2 days a week**	**3–4 days a week**	**5–6 days a week**	**7 days a week**
					

(6) (a) Do you have any food allergies or intolerances? (Please place a tick for your answer in the space provided)

Yes; No.

(7) (b) If Yes, please specify to what foods?

(8) Are you currently trying to lose weight?

Yes; No.

(9) In the 3 previous months have you lost more than 8 kg?

Yes; No.

(10) Do you exercise regularly?

**Frequency of exercise**	**Never**	**Less than once a week**	**1–2 days a week**	**3–4 days a week**	**5–6 days a week**	**7 days a week**
					

(11) Are you currently in athletic training?

Yes; No.

(12) (a) Are you currently taking any prescription drugs, nutritional supplements, or appetite suppressants?

Yes; No.

(13) (b) If so, please specify which ones?

(14) Are you currently suffering from any cardiovascular diseases?

Yes; No.

(15) Are you currently pregnant or breastfeeding?

Yes; No.

(16) Do you have diabetes mellitus, type 1 or type 2?

Yes; No.
