# Supplementary material for: Noodles Made from High Amylose Wheat Flour Attenuate Postprandial Glycaemia in Healthy Adults
Source: Nutrients. 2020 Jul 22;12(8):2171. doi: 10.3390/nu12082171 (PMC7468775; doi:10.3390/nu12082171)
Supplement: Supplementary file 1 [file nutrients-12-02171-s001.pdf]

Table S1. Wheat flour composition (S1).

Noodle Type	Moisture Content (%)	Ash (%)	Protein Content (%)	Starch Amylose Content (%)
LAN27	11.8 (0.4)	0.40 (0.03)	16.2 (0.1)	27 (1.5)
HAN60	11.2 (0.0)	0.45 (0.02)	16.2 (0.21)	60 (1.5)

Values are dry weight basis and mean of duplicates. Values in brackets are SD. LAN, low amylose noodles; HAN, high amylose noodles.

Table S2. Characteristics of the 11 subjects (women = 10, men = 1) in S2 [(a)].

Characteristic	Mean	SEM
Age (year)	22.90	0.91
Weight (kg)	56.66	2.27
Height (m)	1.64	2.40
Body mass index (kg/m^2)	21.00	0.62
Fasting blood glucose (mmol/L)	4.46	0.09

[(a)] Data are expressed as mean ± SEM.

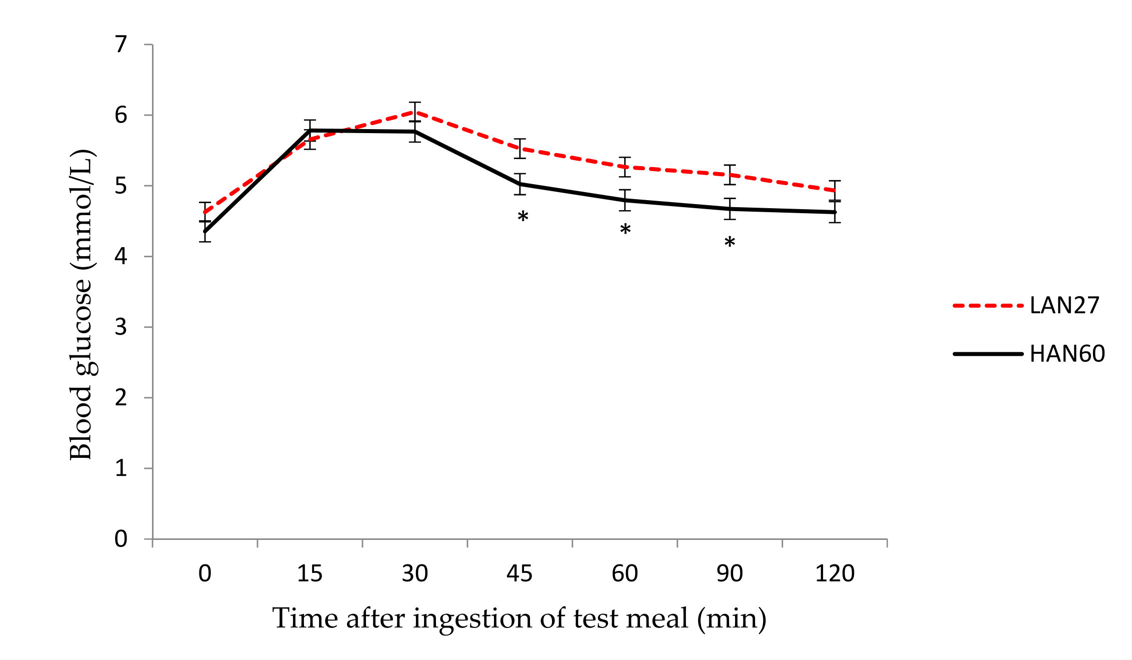

Figure S1. The effects of consuming noodles containing 27.2% and 60% amylose on postprandial glycaemia at different time periods over 120 min. High amylose noodles (60%) induced significantly lower blood glucose at 45, 60 and 90 min ($p < 0.001$). Values presented as mean ± SEM. * indicates statistical significance ($p < 0.05$).

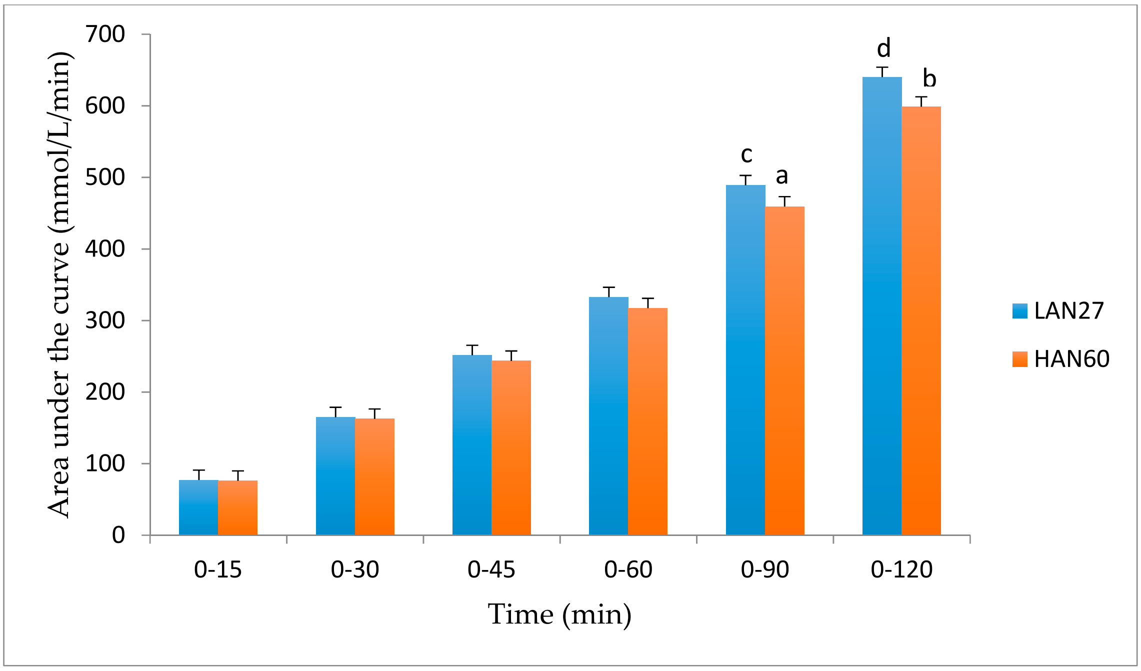

Figure S2. Area under the blood glucose curve over 120 min following the ingestion of test noodles containing 27.2% and 60% amylose. Blood glucose total area under the curve for high amylose noodles (60%) was significantly lower at 90 and 120 min compared to low amylose noodles (27.2%) ($p = 0.021$). Data are presented as mean ± SEM. Difference letters indicate a statistically significant difference ($p < 0.05$).

Secondary statistical analyses were carried to determine the effect of protein on postprandial glycaemia. A secondary analysis was carried out by combining P1 and S1 studies to standardise for amylose and solely examine the effect of protein.

All analyses were conducted using Genstat (version 18.2.0.18409, VSN International, Hemel Hempstead, UK.) Significance was set at $p < 0.05$. Data from the P1 study (12 participants) and the S1 study (11 participants) were combined. The normality of outcome was tested and confirmed as a normal distribution. To examine the effect of the test meal on blood glucose, a blocking factor for individuals and experiment days was used to adjust for the general difference between subjects and experimental days. To ensure test meals had the greatest effect on blood glucose, a linear regression fitting the model into “a score of increasing severity/importance” was done for test meals, exercise and breakfast frequency. An analysis of variance (ANOVA) was then completed for glucose responses to the three test meals. A restricted maximum likelihood (REML) regression model for glucose, to estimate the effects of protein and amylose, was done.
